# Proteins in Soy Might Have a Higher Role in Cancer Prevention than Previously Expected: Soybean Protein Fractions Are More Effective MMP-9 Inhibitors Than Non-Protein Fractions, Even in Cooked Seeds

**DOI:** 10.3390/nu9030201

**Published:** 2017-02-27

**Authors:** Ana Lima, Jennifer Oliveira, Filipe Saúde, Joana Mota, Ricardo Boavida Ferreira

**Affiliations:** Disease & Stress Biology Group, LEAF (Linking Landscape, Environment, Agriculture and Food), Instituto Superior de Agronomia, Universidade de Lisboa, 1349-017 Lisbon, Portugal; jenniferoliveira@live.com.pt (J.O.); fccsaude@gmail.com (F.S.); joana.mota.p@gmail.com (J.M.); rbferreira@isa.ulisboa.pt (R.B.F.)

**Keywords:** soy, soaking and cooking, saponins, polyphenols, proteins, MMP-9, MMP-2, HT-29, cancer

## Abstract

The search for anticancer MMP-9 inhibitors (MMPIs) in food products has become a major goal for research. MMPIs in soy have been related only to saponins and isoflavones, but recently, low specific protein fractions in soybeans were shown to reduce MMP-9 activity as well. The present work aimed at comparing the MMPI potential of protein fractions (P) and non-protein fractions (NP) isolated from soybean seeds, before and after soaking and cooking, mimicking dietary exposures. Reverse and substrate zymography, as well as a fluoregenic DQ gelatin assay were used to evaluate MMP-9 activities. Colon cancer cell migration and proliferation was also tested in HT29 cells. Regarding MMP-9 inhibition, proteins in soy presented IC_50_ values 100 times lower than non-protein extracts, and remained active after cooking, suggesting that proteins may be more effective MMP-9 inhibitors than non-protein compounds. Using the determined IC_50_ concentrations, NP fractions were able to induce higher inhibitions of HT29 cell migration and proliferation, but not through MMP-9 inhibition, whilst protein fractions were shown to specifically inhibit MMP-9 activity. Overall, our results show that protein fractions in soybeans might have a higher role in soy-related cancer prevention as MMPIs than previously expected. Being nontoxic and active at lower concentrations, the discovery of these heat-resistant specific MMPI proteins in soy can be of significant importance for cancer preventive diets, particularly considering the increasing use of soy proteins in food products and the controversy around isoflavones amongst consumers.

## 1. Introduction

Matrix metalloproteinases (MMPs) are a family of zinc-dependent endopeptidases which are involved in the remodeling of connective tissue [1]. One subgroup of MMPs called gelatinases (MMP-2 and MMP-9) has been particularly recognized as important mediators in inflammation [2] and oncologic processes such as tumorigenesis, cell adhesion and metastasis [3,4,5,6,7]. MMP-9 enzymes in particular are highly expressed in colorectal cancer (CRC) and pre-cancer pathologies such as ulcerative colitis and other inflammatory bowel diseases (IBDs) [8,9]. Therefore, it is no surprise that in the last decade the search and development of MMP inhibitors (MMPIs), particularly MMP-9 inhibitors, has become an important branch of research, both in academic as well as in industrial settings. Up to now, numerous MMPIs have been tested in different clinical trials [10] and were already demonstrated to be effective in reducing cancer progression/metastasis and also IBD symptoms [3,8,9,11,12,13,14]. However, targeting MMPs has proven itself difficult due to the fact that MMPs are ubiquitously indispensable for normal development and physiology, and consequently their inhibition induces overall severe adverse side effects [11,15]. In the case of colon cancer or inflammatory bowel diseases, a more convenient and effective approach could be achieved, at least partly, through the long-term ingestion of natural food-born specific MMP-9 inhibitors that may be colon-available. Such can be the case of some legume seeds [16,17,18], among which soy (*Glycine max* L.) has inarguably become the best known for its health-related benefits. Increasingly popular soy has been reported to have bioactivities against cancer [19,20,21] osteoporosis [22,23] and coronary heart disease [24], among others.

As in other legumes, soybean’s anticancer activities are related to phenolic compounds, saponins and phytic acid [25,26,27,28] as well as enzyme inhibitors such as the trypsin and the Bowman–Birk inhibitors [29]. However, the most notable and exclusive anticancer compounds of soy are isoflavones (a class of estrogen-like compounds generally known as phytoestrogens). The peptide lunasin, a small subunit derived from the larger cotyledon-specific 2S seed albumin, also has both anticancer and anti-inflammatory activities [30]. Thus far, soy isoflavones and saponins are the only two bioactive compounds in soy that were shown to be effective MMP inhibitors [31], whilst BBIs were shown to be more likely involved in suppressing the production of superoxide anion free radicals, targeting cancer cells, or suppressing their growth [25,29,32,33,34,35]. Lunasin, according to Kyle et al. [36], does not have a role in MMP inhibition as well, but rather, it involves physical interactions with chromatin and histones [37]. Recently, Lima et al. [18] showed that the protein fraction of few legume seeds, including soybeans can also inhibit MMP-9 in colon cancer cells. This was the first report showing that soy proteins could inhibit MMPs. However, it is still not understood if these proteins have higher MMPI activities than isoflavones or other non-protein compounds. This can be of particular importance for the use of soy in anticancer preventive diets, since legume storage proteins are usually non-toxic and have no anti-nutritional effect, and also because the amount of soyfoods and soy protein additives that are used in the food industry are usually richer in the protein fraction rather than the non-protein fraction.

Under this context, in the present work, we aimed to compare the MMPI activities in the protein and non-protein fractions (polyphenols and saponins) of cooked and uncooked soybeans and to determine which is the most effective to be used in healthier preventive diets. Our main objectives were: (a) to quantify and characterize the presence of these compounds in soy seeds before and after soaking and cooking; (b) to evaluate their effect on MMP-9 activity; (c) to test the effect of these compounds in colon cancer cell migration and proliferation; and (d) to compare protein and non-protein compounds for their potential in preventive diets.

## 2. Materials and Methods

### 2.1. Biological Materials

The biological materials used for the present study were the quiescent seeds of soybean (*Glycine max* L.). The human colon adenocarcinoma cell line, HT29 (ECACC 85061109), obtained from a 44-year-old Caucasian female, was also used throughout this work. HT29 cell lines were maintained in RPMI medium supplemented with 10% (*w*/*v*) of heat-inactivated fetal bovine serum (FBS) and 200 mM glutamine, 2 × 10^4^ UI/mL penicillin and 20 mg/mL streptomycin at 37 °C, in a humidified atmosphere of 5% (*v*/*v*) CO_2_.

### 2.2. Soy Seed Soaking and Heat Treatment

In this work, soy seeds were used dry and cooked, in order to mimic dietary exposures. For dry weight determinations, soy seeds were dried at 50 °C until weight stabilization. For the cooking treatments, seeds were soaked for 24 h at room temperature with deionized water (Mili-Q, Millipore Corporation, Bedford, MA, USA) at a ratio of 1:3 (*w*/*v*). Legume seeds were then cooked in deionized water in a ratio of 1:20 (*w*/*v*) in a metal container on a hot plate until they reached a soft texture [38]. In both cooked and uncooked seeds, the protein and non-protein fractions were extracted for the subsequent analysis.

### 2.3. Extraction and Quantification of Soluble Proteins

Legume seeds (uncooked and cooked) were ground in a Bimby^®^ TM 31 cooking robot until a fine and homogeneous powder was obtained. For protein extractions samples were mixed with polyvinylpolypyrrolidone (PVPP) in a portion of 1:4 (*w*/*w*), and extracted with 100 mM Tris HCl buffer at pH 7.5, in a ratio of 1:5 (*w*/*v*) as described earlier [19]. This solution was kept stirring overnight at 4 °C. Samples were then centrifuged in a Beckman J2-21M/E centrifuge at 12,000× *g* for 1 h at 4 °C, and the supernatant was collected and then stored at −20 °C for further analysis. Protein quantification was performed according to Bradford [39].

### 2.4. Extraction and Quantification of Non-Protein Compounds

Non-protein compounds were extracted according to the method described by Makkar and Becker [40]. Samples were ground in a Bimby TM 31 cooking robot, as described in 2.3 and 80% (*v*/*v*) methanol was added in a 1:20 (*w*/*v*) portion, and stirred overnight. The contents were centrifuged at 3500× *g* in a Beckman Coulter™ Allegra™ 25R centrifuge for 10 min and the supernatant collected. The precipitate was washed three times with 80% (*v*/*v*) methanol in a portion of 1:10 (*w*/*v*), centrifuged at 3500× *g* in a Beckman Coulter™ Allegra™ 25R centrifuge for 10 min, and the supernatant was collected. The supernatant was then evaporated at 50 °C until dry.

#### 2.4.1. Saponin Quantification

Total saponin content was quantified by the method described by Hiai et al. [41] using soybean saponins as standard. To the extracts, a solution with 8% (*v*/*v*) of 1:5 (*v*/*v*) was added in a ratio of 1:4 (*v*/*v*), followed by a solution with 80% (*v*/*v*) of vanillin in ethanol and finally 1:50 (*v*/*v*) of 72% (*w*/*v*) sulfuric acid. The mixture was allowed to stir in a water bath at 0 °C. Subsequently, the mixture was heated in a bath with water at 60 °C for 10 min, and placed again in cold water until cool. Absorbance was read at 544 nm on a Syenery HT spectrophotometer, Bio-TEK.

#### 2.4.2. Polyphenolic Compounds

Polyphenolic compounds were quantified by the Folin–Ciocalteau reagent using gallic acid as standard. The lyophilized powder was treated with 10 µL of 70% (*v*/*v*) acetone, 10 µL of 0.5% (*v*/*v*) acetic acid and 80 µL of 7% (*w*/*v*) sodium carbonate. Subsequently, a volume of 100 µL of Folin–Ciocalteau was added and the mixture was vortexed. The solution (200 µL) was incubated for 8 min at room temperature and the absorbance was read in a Syenery HT Bio-TEK spectrophotometer at 765 nm [42].

### 2.5. Half Maximal Effective Inhibition of MMPs (IC_50_)

MMP-9 IC_50_ values were assessed in sterile 96-well plates (Greiner Bio-one, Germany), using the micro dilution method as described before [43]. Briefly, 50 μL of RPMI medium was added to each well. Then, concentrated samples (protein or non-protein extracts) were added to the first well and serially diluted 1:2 to each adjacent well, up to 10 dilutions. Subsequently, 50 μL of the HT29 cell suspension with a concentration of 2 × 10^5^ cells/mL was added to each wells. A positive control (50 μL RPMI medium + 50 μL cell suspension) and a negative control (100 μL RPMI medium) were performed. After 48 h growth, MMP-inhibition was tested using the DQ gelatin assay as described in Lima et al. [18]. The fluorogenic dye-quenched (DQ)-gelatin substrate was purchased from Invitrogen (Carlsbad, CA, USA) and dissolved in water at 1 mg/mL. All solutions and dilutions were prepared in assay-buffer (50 mM Tris-HCl buffer, pH 7.6, containing 150 mM NaCl, 5 mM CaCl_2_ and 0.01% *v*/*v* Tween 20). A 96-well micro-assay plate (chimney, 96-well, black) was used and each well was loaded with 100 μL of extracellular HT29 media (containing MMP-9 and MMP-2) after exposure to the different concentrations of the soy extracts. Subsequently, DQ-gelatin (at a final concentration of 2.5 μg/mL) was added to each well (for a final volume of 200 μL) and the plate was allowed to incubate again, for 1 h. In the presence of active gelatinolytic activity the DQ-gelatin substrate becomes fluorescent. Fluorescence levels were measured (excitation 485 nm/emission 530 nm). After plotting, the value correspondent to a 50% gelatinase inhibition was selected as the IC_50_ for each fraction.

### 2.6. Specific MMP-9 Inhibition

In order to evaluate specific MMP-9 inhibition, the DQ gelatin assay was used once more, as described in 2.5, but with some adaptations. Each well was loaded with 0.1 mM (for a final volume of 200 μL) MMP-9 (Sigma). Protein or non-protein extracts were added to the MMP-9 solution, in their respective calculated EC_50_ concentrations (for a final volume of 200 μL), and the plate was incubated for 1 h at 37 °C to allow inhibition. Subsequently, in order to quantify MMP-9 activity, DQ-gelatin (at a final concentration of 2.5 μg/mL) was added to each well and the plate was allowed to incubate again, for 1 h. Fluorescence levels were measured (excitation 485 nm/emission 530 nm). In each experiment, both positive (no protein fraction) and negative (no enzyme) controls were included for all samples, to correct for possible proteolytic activities present in the soy extracts. All data were corrected by subtraction of their corresponding negative controls.

### 2.7. Sodium Dodecyl Sulfate-Polyacrylamide Gel Electrophoresis

Cooked and uncooked soy samples were treated with 100 mM Tris-HCl buffer, pH 6.8, containing 100 mM β-mercaptoethanol, 2% (*w*/*v*) SDS, 15% (*v*/*v*) glycerol and 0.006% (*w*/*v*) m-cresol purple, and heated at 100 °C for 5 min. One-dimension electrophoresis was carried out, following the method described by Laemmly et al. [44] in a 12.5% (*w*/*v*) acrylamide resolving gel and a 5% (*w*/*v*) acrylamide stacking gel, and performed in a vertical electrophoresis unit at 100 V and 20 mA per gel. Gels were fixed for 20 min in 10% (*w*/*v*) TCA, and stained in 0.25% (*w*/*v*) Coomassie Brilliant Blue R-250, 25% (*v*/*v*) 2-propanol and 10% (*v*/*v*) acetic acid. Destaining was carried in a solution of 25% (*v*/*v*) 2-propanol and 10% (*v*/*v*) acetic acid.

### 2.8. Reverse Gelatin Zymography

Reverse zymography was used to detect and quantify MMPI proteins in the protein fractions of cooked and uncooked seeds as described in Hawkes et al. [45], with some modifications. Protein samples were treated with zymographic buffer (313 mM Tris-HCl buffer, pH 6.8, containing 10% (*w*/*v*) SDS, 50% (*v*/*v*) glycerol and 0.05% (*w*/*v*) bromophenol blue) and loaded in SDS-polyacrylamide (12.5% *w*/*v* acrylamide) gels copolymerized with gelatin (1% *w*/*v*) and 1 µmol/mL MMP-9. Electrophoresis was performed as described in 2.5 and the gels were washed three times in 2.5% *v*/*v* Triton X-100, for 60 min each, to remove SDS. Gels were then incubated overnight at 37 °C, with developing buffer (50 mM Tris-HCl buffer, pH 7.4, containing 5 mM CaCl_2_, 1 µM ZnCl_2_ and 0.01% *w*/*v* sodium azide), stained with Coomassie Brilliant Blue G-250 0.5% (*w*/*v*) in 50% (*v*/*v*) methanol and 10% (*v*/*v*) acetic acid for 30 min, and destained with a solution of 50% (*v*/*v*) methanol, 10% (*v*/*v*) acetic acid in water. Dark bands visible against a white background marked the MMPI-mediated inhibition of gelatin degradation [45].

### 2.9. In Vitro Colon Cancer Cell Assays

#### 2.9.1. Cell Migration Assay

For cell migration analysis, the wound healing assay was performed. HT29 cells (5 × 10^5^ cells/well) were seeded in 6-well plates and allowed to reach to 80% confluence. Wounds were performed by making a scratch across the cell monolayer to create an open gap, mimicking a wound. Cells were then washed twice with PBS to remove floating debris. Each well was subsequently filled with fresh media containing the samples under study, in a concentration of 100 μg/mL and allowed to grow for 48 h. The invaded area after 48 h was calculated in each treatment and compared to the initial area at 0 h, to determine the area covered by migrating cells into the denuded zone at the beginning of treatment. This comparison allowed us to assess the inhibitory effect (if any) exerted by each fraction, on the HT29 cell migrating capacity.

#### 2.9.2. Cell Proliferation Assay

HT29 cultured cells were seeded on 96-well plates (2 × 10^4^ cells/well) and soy extracts were added to the growth media in different concentrations, and incubated for 24 h. After each treatment, the extracellular media was collected, and the wells were washed with PBS to remove unattached cells. Cell proliferation and viability was determined using the standard 3-(4,5-dimethylthiazol-2-yl)-2,5-diphenyltetrazolium bromide (MTT) assay as described by Carmichael et al. [46].

### 2.10. Substrate Gelatin Zymography

To determine metalloproteinase activities in HT29 cancer cell culture supernatants, a gelatin-zymography was performed according to standard methods [47], as described before but SDS-polyacrylamide gels (12.5% *w*/*v* acrylamide) were copolymerized with 1% (*w*/*v*) gelatin. The cell culture supernatants were treated with a non-reducing buffer (62.6 mM Tris-HCl buffer, pH 6.8, containing 2% *w*/*v* SDS, 10% *v*/*v* glycerol and 0.01% *w*/*v* bromophenol blue) and loaded into each well. After electrophoresis, gels were washed three times in 2.5% (*v*/*v*) Triton X-100 for 90 min each to remove the SDS, incubated overnight with developing buffer and stained with Coomassie Brilliant Blue G-250 as described above for reverse zymography. White bands visible against a blue background marked the gelatinase activity of each proteinase [47].

### 2.11. Statistical Analysis

All experiments were performed in triplicate at least three independent times, and the data are expressed as the mean ± standard deviation (SD). SigmaPlot software (version 12.5) was used for comparing different treatments, using one-way and two-way analysis of variance (ANOVA). Tukey’s test was used to compare differences between groups and the statistical differences with *p* value less than 0.05 where considered statistically significant.

## 3. Results and Discussion

Currently, soybeans and soy-foods have become extremely popular in the food industry as a health staple [48], with numerous observational and clinical studies supporting their claims [49,50]. Although isoflavones and saponins are highly effective bioactive compounds, they can induce secondary effects [51,52] that cause a lot of controversy in consumers. The recent discovery of a protein fraction in soy with anticancer potential [18] may alter this view of soy-foods. In this work, we compared the MMP inhibitory activities in the protein and non-protein fractions from soy.

Surprisingly, most studies testing anticancer activities in legumes use either uncooked seeds, or the isolated bioactive compounds, such as the isoflavone genistein [18,31]. However, in order to improve the nutritional quality of soy-foods, inhibitors such as lectins are generally inactivated by soaking and heat treatment during food processing. Since cooking and soaking is known to reduce or destroy the nutritional compounds in foods, in a more realistic perspective, the consumption of soy-foods and soy-related products might have a lesser effect in MMP activities. In order to use soy in MMPI-related cancer preventive diets, it becomes important to test its activities in cooked seeds. This is of significant importance to understand its impact in consumer’s health. Hence, in this work, extractions were carried out with the whole seed, including the tegument and were soaked and cooked. To our knowledge, there are no reports comparing these types of activities in cooked and uncooked seeds.

### 3.1. Cooking Affects the Amounts of Bioactive Compounds in Soy Seeds

In the present work, we extracted the protein and non-protein fractions in soybeans and quantified the amounts of potentially bioactive compounds before and after soaking and cooking. Previous works have shown that the most prominent bioactive compounds in soy are saponins, polyphenols and proteinase inhibitors. Saponins are glycosides which are usually considered undesirable in food products because of their toxicity and hemolytic activity [52], but have been reported to have anticancer [53,54,55,56,57] and anti-inflammatory [58] effects, in in vitro and in vivo assays [59,60]. Phenolic compounds on the other hand are usually pointed out as the main responsible for the antioxidant properties of soy [20,21,22,25]. Isoflavones are inarguably the most active ones, exhibiting several biological activities against several pathologies, including as osteoporosis, cardiovascular diseases and menopausal symptoms as well as cancer [61,62,63]. The anticancer effects of isoflavones have unquestionably been most extensively studied in breast cancer [64], but less in colon cancer. As for the protease inhibitors, which are part of the Kunitz family and Bowman–Birk family, some of these peptides are known to have anti-inflammatory effects [65,66,67] and are effective in inhibiting carcinogenesis after induction of radiation and chemical treatment [25]. However, the mechanism responsible for this effect is still poorly studied.

The total amounts of saponins, polyphenols and proteins in each fraction (protein and non-protein) extracted from cooked and uncooked soybean seeds are expressed in Table 1.

It is widely known that food processing improves flavor and palatability, but also increases the bioavailability of nutrients by inactivating the antinutritional factors [52]. Many nutrients are heat-sensitive, such as some proteins. Saponins, on the other hand, are known to be more heat stable, but their concentration can be fairly reduced by soaking, germination and/or fermentation [62]. Previous in vitro studies suggest that cooking increases the digestibility of protein and starch reduction of trypsin inhibitors, with no change in mean nutritional quality of these macronutrients [68,69], although it may alter the natural antioxidant content in food [70,71].

According to Table 1, it seems clear that after soaking and cooking all metabolites were very significantly reduced (*p* < 0.05) to less than 50% of their initial content. Saponins were reduced by 57.6%, whilst polyphenols presented a 60% reduction. As expected, protein content was the most affected after cooking, with a 94% reduction (*p* < 0.05). Although some of these compounds are thermoresistant as discussed before, previous works have also determined that in soy structural phenolic compounds can be released by thermal depolymerization during cooking [72] or by washing out during soaking [73], which is consistent with our results. In order to understand if these cooking-induced depletion in bioactive compounds could reduce the anticancer potential of soy we further set out to determine if the cooking process affects or not MMPI activities in both fractions.

### 3.2. Proteins in Soy are Better MMP Inhibitors Than Polyphenols and Saponins

Since MMP-9 inhibitors (MMPIs) are considered anti-angiogenic agents and metastasis deterrents for colorectal cancer, and have also been demonstrated to effectively inhibit pre-cancer states such as colitis and other inflammatory bowel diseases [74]. In order to compare the MMP inhibitory potential of the protein and non-protein extracts from soybean seeds, and to select a concentration to use in the subsequent studies, we determined the half maximal effective inhibition of MMPs (IC_50_) values for soluble protein (P) and non-protein (NP) fractions. IC_50_ values were found to be of 91.6 ± 10 μg DW/mL for protein fractions and 10.8 ± 0.02 mg DW/mL for phenolics and saponin extracts. These IC_50_ levels substantiate that both protein and non-protein fractions can indeed reduce MMP activities, but they varied very significantly between them (*p* < 0.001), being 100 times lower in the protein fractions than in the non-protein fractions. These orders of magnitude are consistent with previous works. For instance, Lima et al. [18] found that 100 g/mL of soy albumin extract reduce 95% of MMP activity, whereas Xu and Cheng [28] showed that up to 5 mg/mL of polyphenolic extracts from soy are required for antioxidant and anti-proliferative activities in cancer cells. In this work, IC_50_ values seem higher than previous works [18], which could be explained by the fact that we used whole seeds with teguments. Nonetheless, the large difference between the IC_50_s determined for protein fractions and the non-protein fractions clearly suggests that protein MMPIs in soy might be better inhibitors than polyphenols and saponins. Hence, our results suggest that daily intake of soy protein MMPIs may be of potential importance for cancer preventive diets, especially in colorectal cancer and colon-related inflammatory diseases, where these compounds can exert their effect without being absorbed to the bloodstream.

### 3.3. Cooking Affects MMP-9 Inhibitory Activities of Non-Protein Compounds, but Not of MMPI Proteins

**Figure 1:**
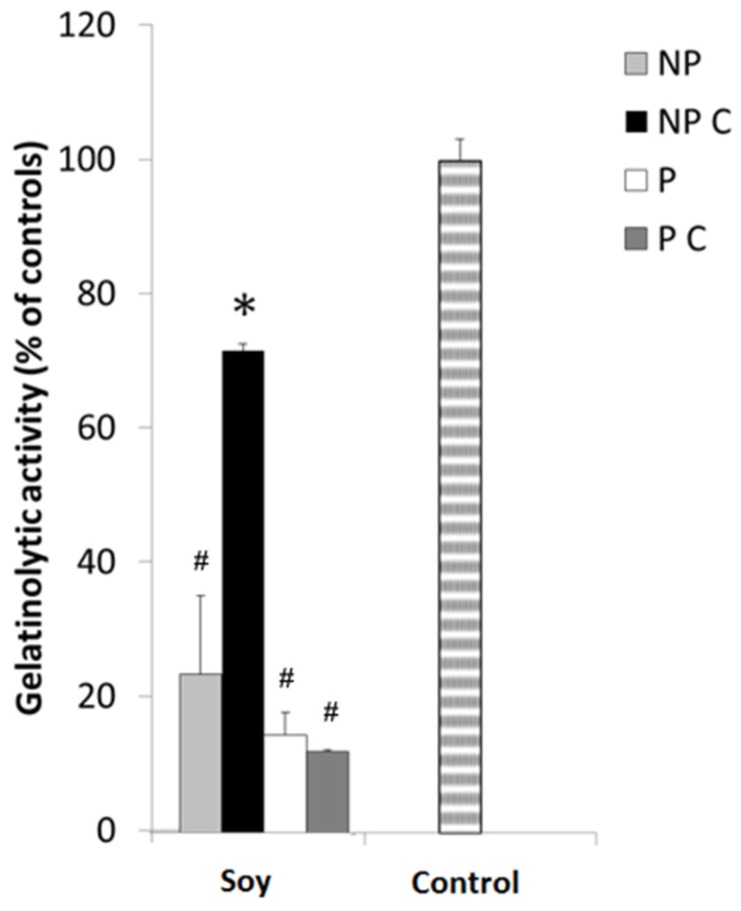
The effect of protein and non-protein fractions isolated from cooked or uncooked seeds of Glycine max on the proteolytic activity of MMP-9. Non-protein uncooked fraction (NP), non-protein cooked fraction (NP C), protein uncooked fraction (P) and protein cooked fraction (P C). The control does not inhibit MMP-9, resulting in 100% proteolytic activity for this protease. Protein fractions, cooked (P C) and uncooked (P), were added at a concentration of 100 µg/mL and non-protein fractions, cooked (NP C) and uncooked (NP) were added at a concentration of 10 mg/mL. Gelatinolytic activity was measured by the DQ fluorogenic assay. MMP activities are expressed as relative fluorescence as a percent of controls, and represent the averages of at least three replicate experiments (*n* = 3) ± SD. ∗ *p* < 0.05 between samples and # *p* < 0.05 in relation to controls.

An estimated 3.45 million new cases of cancer and 1.75 million deaths from cancer occurred in Europe in 2012 [75], with colorectal cancer (CRC) being the second most common cause of cancer death in the European Union [75,76] despite the intensive research made and the significant advances in diagnosis, screening and treatment [77,78]. Several studies point to the fact that MMP activity inhibition appears to be a promising anticancer therapy [10,79]. Within MMPs, MMP-9 is the most studied because of their importance in cell migration, angiogenesis and metastasis formation [33,80]. Hence, we further set out to analyze the extracts’ specific activity towards MMP-9 enzymes, in their determined IC_50_ concentrations, using the DQ-gelatin assay. Results are presented in Figure 1 and are expressed as percent of MMP-9 activity in relation to controls.

Results show that indeed all uncooked extracts were able to inhibit MMP-9, as expected by the IC_50_ levels, however, after soaking and cooking, only the protein extracts maintained their activities, whilst soy NP compounds were significantly reduced (*p* < 0.05). These results substantiate the data in Table 1, suggesting once again that the protein fraction in soy might hold a higher potential for MMPI diets in more realistic scenarios.

**Figure 2:**
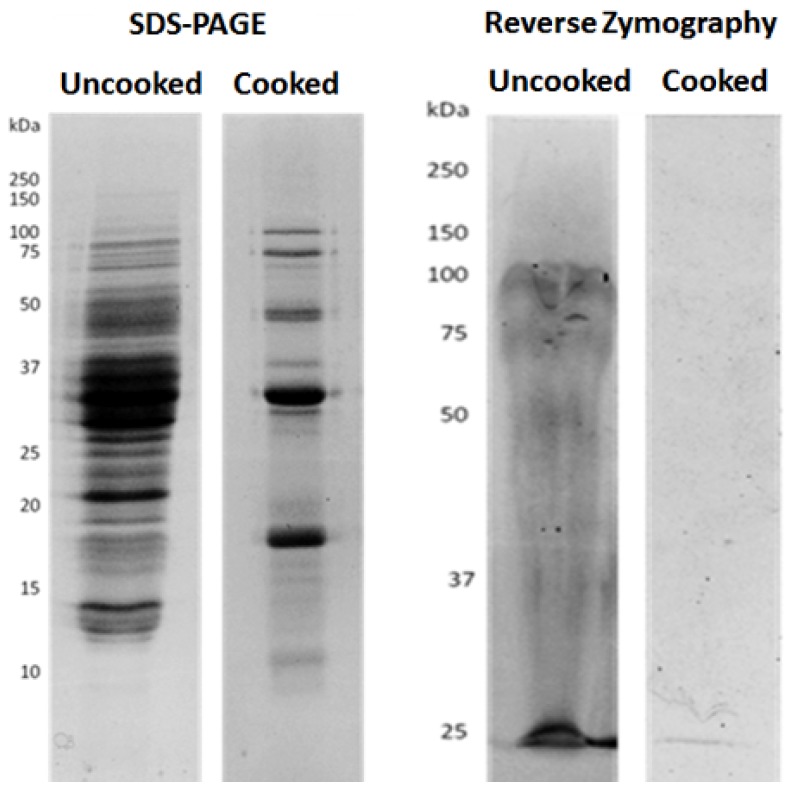
Cooked and uncooked protein extracts present MMPI activity. Representative images of the polypeptide distribution between Glycine max seeds uncooked or cooked, and visualized by: SDS-PAGE (**left**); or by reverse gelatin zymography (**right**). Protein extracts (100 µg/mL) were loaded onto 17.5% (*w*/*v* acrylamide) polyacrylamide gels, copolymerized with gelatin and MMP-9 in the case of reverse zymography.

It is known that protease inhibitor peptides are stable within reasonable limits, at elevated temperatures, and some proteins of lower molecular weight and some leguminous species also present thermostability, which could be the case of soybean protein fractions [71]. Through reverse zymography, as shown in Figure 2, we could pinpoint which proteins showed a capacity for inhibition or resistance to MMP-9, and which were resistant to cooking. In this technique, proteins are electrophoretically separated in an acrylamid gel co-polymerized with MMP-9 and its substrate gelatin. Hence, after incubation, the gelatin in the gel suffers MMP-9-induced proteolysis, resulting in a clear gel. The observed remaining visible bands represent the proteins which are resistant to or inhibit MMP-9 activity.

Figure 1 shows, as expected from Table 1, that soybean presented a high loss of protein after cooking. In addition, reverse zymographies show the existence of some polypeptides with MMPI activity in crude seeds, which is consistent with previous works [18]. However, after cooking, only one polypeptide band, with approximately 25 kDa, could inhibit MMP-9. This protein band may be one or more specific low molecular mass protein(s) with MMPI activities. In soybeans as well as other legumes, various proteins and peptides such as lunasin, and protease inhibitors, ranging from 4 kDa to 22 kDa have been largely studied [30,81,82], but until now they were not related to MMP inhibition. This is the first report, to our knowledge, of a protein MMPI from soy that is also heat resistant. Since the DQ-gelatin as well as the zymographic analysis incubate directly the MMP-9 enzymes with the extracts, it seems plausible to infer that these MMPIs directly bind to the enzyme, hence blocking its activity. This is consistent with its protease inhibitory activities referred to in other studies [81,82].

### 3.4. Soy Polyphenols Reduce Cancer Cell Migration Even without Inhibiting MMP-9

Since the earliest work on the importance of MMPs in cancer, there has been a clear relation between the inhibitory effect on MMP with its ability for extracellular matrix degradation and cancer cell migration [18]. Several studies have linked the inhibition of MMPs to a corresponding inhibition of cell invasion in standard wound healing assays [79]. In this assay, the ability of cells to invade a gap cut in the monolayer of the cells allow an indirect measurement of their ability to metastasize and of their MMP-9 activity [80].

**Figure 3:**
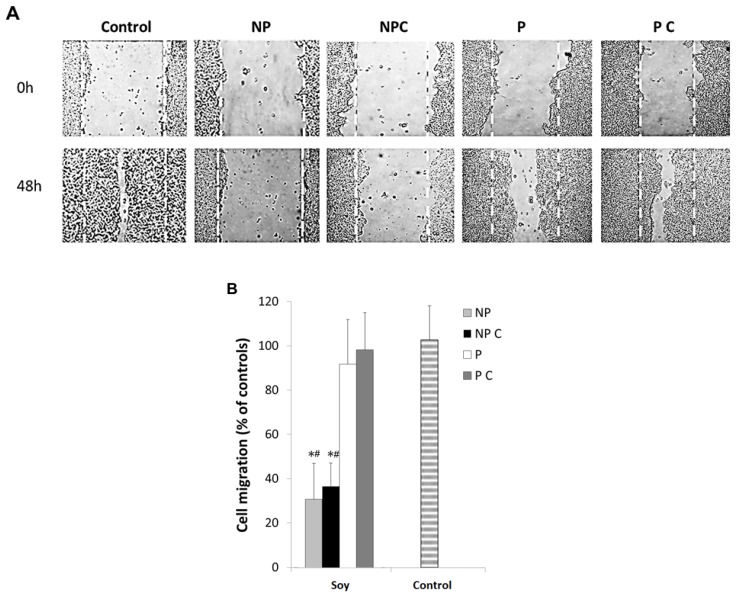
HT29 cell migration after exposure to non-protein uncooked fraction (NP), non-protein cooked fraction (NP C), protein uncooked fraction (P) and protein cooked fraction (P C), as determined by the wound healing assays. (**A**) Representative images of cell migration showing the inhibitory effect of different fractions of Glycine max seeds on HT29 cell migration. Cells were grown until reaching 80% confluence and the monolayer was scratched with a pipette tip (0 h). Cells were then exposed to 100 µg protein/mL, cooked (P C) and uncooked (P), and to 10 mg non-protein/mL, cooked (NP C) and uncooked (NP) and cell migration was recorded after 48 h; (**B**) Relative migration rates. Values are the means of at least three replicate experiments ± SD, and are expressed as a percent the wound closure in relation to 0 h. * represents *p* < 0.05 between samples and # represents *p* < 0.05 when compared to controls.

Here, the studied extracts were further analyzed for their effectiveness in reducing cancer cell migration proliferation in HT29 cells. Figure 3A,B shows cell migration patterns of HT29 cells after a 48 h exposure to P and NP fractions, both in cooked as well as uncooked seeds using the wound healing assay.

Results clearly demonstrate that the different fractions of soybeans had an inhibitory effect on cell migration, but significant differences (*p* < 0.05) were observed between the protein and non-protein fractions. Under their IC_50_ concentrations, in general, cells treated with non-protein compounds presented significantly (*p* ≤ 0.05) lower cell migration after 48 h of incubation compared to the controls (Figure 3B), which was significantly higher in cooked cells (*p* < 0.05). Previous works showed that saponins and phenolics can induce these inhibitions. Kang et al. [31] found that soybean saponins inhibited cell migration in both HT29 cells and fibroblast cancer cells (HT1080 cells) at a concentration of 30 to 300 μg/mL. Polyphenols present in foods have also been shown to have antitumor activity by inhibiting cell migration, MMP secretion, cell growth and cell adhesion and further induce apoptosis [83], which is in agreement with our results. However, in comparison, the proteins fractions, which had induced a similar MMP-9 inhibition, did not significantly reduce cell migration. This seems to suggest that the MMP-9 inhibition induced by this concentration of the protein fractions is not enough to reduce cell migration. Indeed, we know from previous reports that soy proteins can reduce cancer cell migration in concentrations of 100 μg protein/mL [18]. However, in previous works, this concentration inhibited almost completely MMP-9 activities, which is not the case here, since we used IC_50_ concentrations. It is plausible that a 50% inhibition in total gelatinases in the extracellular media is not enough to fully reduce cell migration. Nonetheless, it is important to highlight that the protein fraction was 100 times lower than the phenolic and saponin fraction, hence these results do not suggest that proteins are less effective in reducing cancer cell migration. It suggests, however, that the effect exerted by non-protein compounds in HT29 migration are not dependent on MMP-9 inhibition as the protein effects probably are. We further set out to determine if the inhibitions observed in cancer cell migration were related to any type of cytotoxicity or to specific MMP-9 inhibition.

### 3.5. Cancer Cell Proliferation

**Figure 4:**
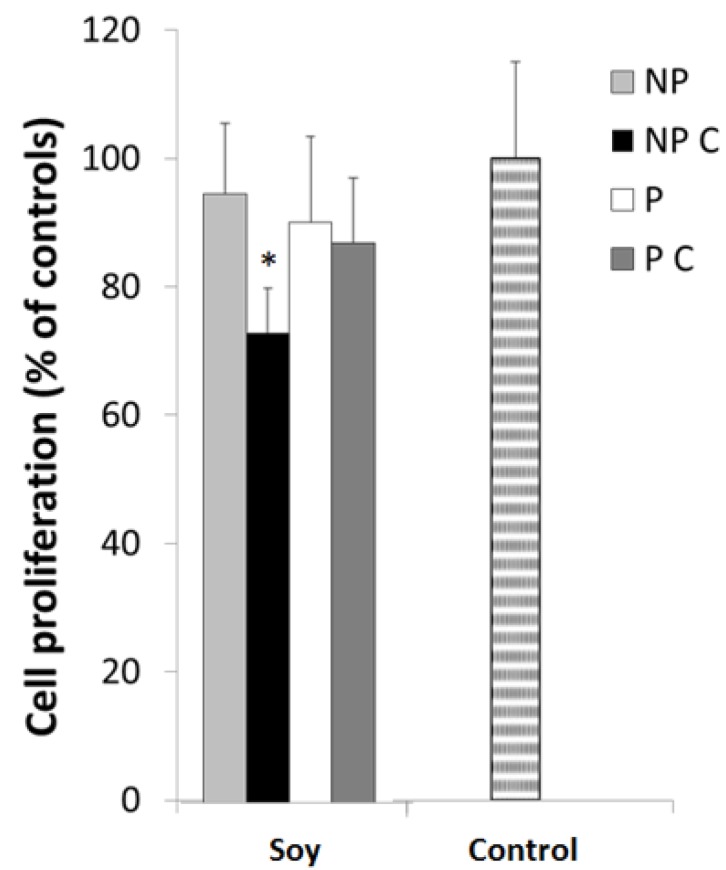
HT29 cell proliferation after a 24 h exposure to non-protein uncooked fraction (NP), non-protein cooked fraction (NP C), protein uncooked fraction (P) and protein cooked fraction (P C). Cells were grown for 24 h in the presence of 100 µg protein/mL, cooked (P C) and uncooked (P) fractions and to 10 mg non-protein/mL, cooked (NP C) and uncooked (NP) fractions and stained with MTT. Values represented are the means of at least three replicate experiments (*n* = 3) ± SD and are expressed as a percentage of the control. * represents *p* < 0.05 between samples.

In order to test the cytotoxic effects of the determined IC_50_ concentrations, we evaluated the effect of the studied fractions on HT29 cell viability and proliferation (Figure 4).

Overall, results demonstrate that most of the extracts did not show cytotoxicity to the cells, since cell viability was maintained, although after cooking, non-protein compounds soybean induced a significant inhibition (*p* < 0.05) in cell proliferation, suggesting saponins or phenolic compounds may exert toxic effects to the cells. Indeed, previous studies have shown greater cytotoxic effects induced by saponins and phenolics. For example, Fereidunian et al. [33] found that 200 μg/mL of soybean BBI did decrease cell multiplication at a concentration of 400 μg/mL in HT29 cells. However, these works involved specific isolated molecules, and in higher concentrations. The fact that the extracts tested here induced greater effect on the cell migration, and not on cell multiplication may also be associated to their mechanism of inhibition. One of the factors most associated with the reduction of cell migration rate is the activity of MMP-2 and MMP-9. Thus, in this work, the gelatinolytic activity of the HT29 cells extracellular medium was further analyzed after exposure to different NP and P fractions.

### 3.6. Soy Extracts Inhibit MMP-9 in HT-29 Extracellular Media, but Not MMP-2

**Figure 5:**
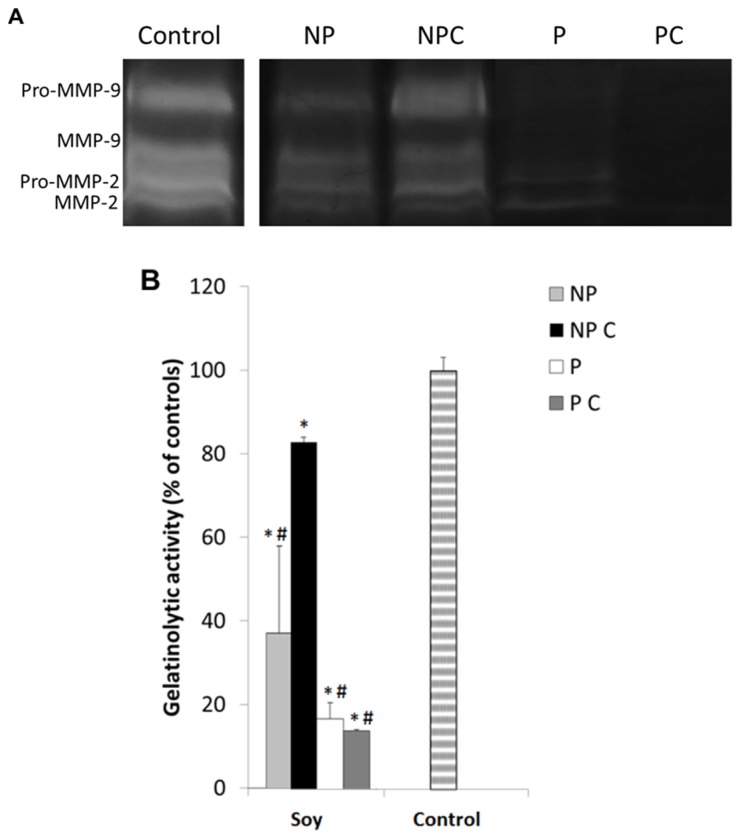
(**A**) Representative image of the zymographic profiles of MMP-9 and MMP-2 activities in presence to different cooked and uncooked fractions isolated from soy seeds. MMP-9 and MMP-2 proteolytic activities were detected in HT29 extracellular media after a 48 h exposure to 100 µg protein/mL cooked (P C) and uncooked (P) fractions and 10 mg non-protein/mL cooked (NP C) and uncooked (NP) fractions isolated from Glycine max seeds. HT29 extracellular media were loaded in 12.5% (*w*/*v* acrylamide) polyacrylamide gels co-polymerized with 1% (*w*/*v*) gelatin; (**B**) Proteolytic activity of gelatinases present in the HT29 extracellular media after a 48 h exposure to 100 µg protein/mL cooked (P C) and uncooked (P) fractions and to 10 mg non-protein/mL cooked (NP C) and uncooked (NP) fractions isolated from Glycine max seeds, as quantified by the DQ fluorogenic method. Results are expressed as relative fluorescence as a percent of controls and represent an average of at least three replicate experiments (*n* = 3) ± SD. * represents *p* < 0.05 between samples and # represents *p* < 0.05 compared to controls.

Under normal conditions, cancer cells secrete active MMP-2 and MMP-9 into the extracellular medium and upon degradation of the surrounding environment, they are able to proceed with cell migration [3,80], and consequently with the formation of metastases. The specificity of MMP inhibition may be of significant importance in this matter, because it may maintain low secondary effects and also induce higher efficacy in prevention or treatment. The inhibition of MMP-2 or MMP-9 in the HT29 cells exposed to the different soy extracts was analyzed through zymographic analysis as well as the DQ gelatin assay. Results can be observed in Figure 5A,B.

Both gelatinolytic activities show a significant decrease (*p* ≤ 0.05) in the presence all the studied fractions, which is consistent to the previous results and to the cell migration inhibition. However, although non-protein fractions were more effective in reducing cell migration, soluble proteins (both uncooked and cooked) showed greater ability to inhibit gelatinolytic activity when compared to the non-protein compounds (*p* ≤ 0.05) inhibiting more than 80% of MMP activity. In fact, non-protein extracts were much less efficient in inhibiting both gelatinases. These results corroborate that soy proteins are indeed more efficient in inhibiting MMPs than non-protein compounds. On the other hand, the fact that the non-protein compounds did not inhibit MMPs but reduced multiplication and invasion substantiates that they may be associated with another anticancer mechanism besides gelatinase inhibition, such as in the decrease of reactive species of oxygen [83], or others.

Moreover, zymographic results also show that in general, soy protein extracts were much more effective in inhibiting pro-MMP-9 (92 kDa) and active MMP-9 (83 kDa), than the pro-MMP-2 and MMP-2. It is known that MMP inhibitors may have an action at different molecular levels: (i) inhibition of transcription; (ii) inhibition of post-transcription; (iii) inhibition of translation; (iv) post-translational degradation; and (v) inhibition of secretion or activity [33]. In this case, inhibition of active MMP-9 may have been achieved either by a direct inhibition of MMP-9 expression or activity, since there was also a decrease in pro-MMP-9. Either way, because most of the problems involved with MMP inhibitors are related to their lack of specificity [11,15], the finding of specific MMP-9 inhibitors in soy can be of significant importance for our understanding of the anticancer and anti-inflammatory mechanisms in this legume species.

## 4. Conclusions

Overall, our work shows that protein fractions in soybeans might have a higher role in soy-related cancer prevention and reduction than previously expected. First, we showed for the first time that the protein fractions of soy seeds are more effective and selective MMP-9 inhibitors than the non-proteins compounds. Furthermore, they remain active after cooking. Finally, they are more specific inhibitors of MMP-9. The discovery of heat-resistant specific MMPI proteins in soy can be of significant importance for cancer preventive diets, particularly considering the increasing use of soy proteins in food products. However, mostly, with all the controversy around the consumption of soy isoflavones in diets, our results open doors to developing novel strategies using soy in cancer preventive diets without consumer concerns regarding potential health risks of polyphenols and saponins.

## Figures and Tables

**Table 1:** Quantification of bioactive compounds saponins, polyphenols and soluble proteins in uncooked (UC) and cooked (C) fractions of Glycine max seeds. Results represent an average of at least three replicate experiments (*n* = 3) ± SD. ^a^ represents a significant difference (*p* < 0.05) between cooked and uncooked seeds.

	Saponins mg/g DW	Phenolics mg/g DW	Proteins mg/g DW
UC	3.6 ± 0.24	1.7 ± 0.16	297.4 ± 8.65
C	1.6 ± 0.24	0.7 ± 0.06	15.7 ± 4.82
% of lost	57.6 ^a^	60.9 ^a^	94.7 ^a^
